# Exogenous Testosterone Increases Decoy Effect in Healthy Males

**DOI:** 10.3389/fpsyg.2018.02188

**Published:** 2018-11-13

**Authors:** Jiajun Liao, Yang Zhang, Yingchun Li, Hong Li, Samuele Zilioli, Yin Wu

**Affiliations:** ^1^Shenzhen Key Laboratory of Affective and Social Cognitive Science, Shenzhen University, Shenzhen, China; ^2^College of Psychology and Sociology, Shenzhen University, Shenzhen, China; ^3^School of Science, Harbin Institute of Technology (Shenzhen), Shenzhen, China; ^4^Department of Psychology, Wayne State University, Detroit, MI, United States; ^5^Department of Family Medicine and Public Health Sciences, Wayne State University, Detroit, MI, United States

**Keywords:** androgen, human male, decoy effect, preference, decision-making

## Abstract

There is increasing interest in the role played by testosterone in economic decision-making and social cognition. However, despite the growing body of findings in this field of research, no empirical study to date has tested whether testosterone modulates decision-making when an asymmetrically dominated decoy option is introduced in a choice set. Within a choice set that comprises two options, an asymmetrically dominated decoy option is a third option that, when introduced in the choice set, is much worse than one of the existing options, but comparable to the other existing option. Introduction of a decoy option leads to a preference toward the dominating option (i.e., decoy effect). Healthy male participants (*n* = 63) received a single-dose of 150 mg testosterone gel in a double-blind, placebo-controlled, between-subjects design. At 180 min post-administration, participants took part in a decision-making task to elicit decoy effect. Results showed that participants in the testosterone group made less consistent choices and more target choices (i.e., decoy effect) than participants in the placebo group. These findings are interpreted in light of the dual-process theory and are in line with existing evidence suggesting that testosterone promotes more intuitive and automatic judgments in human decision-making.

## Introduction

Testosterone is a sex steroid that, in addition to being involved in reproductive physiology and morphology, plays an important role in various psychological processes, including decision-making (Eisenegger et al., 2011). Higher testosterone levels have been associated with dominant behaviors, such as social aggression (Eisenegger et al., 2011) and risk-taking (Apicella et al., 2015), but also prosocial acts (van Honk et al., 2012). Recent evidence has reconciled these contrasting findings by invoking the moderating role played by contextual influences (Boksem et al., 2013; Dreher et al., 2016). Although the realization that testosterone influences decisions in a context-dependent fashion is relatively new, the broader idea that human choices are fine-tuned to the context in which options are presented has long been known (Kahneman, 2011). The “framing effect,” the idea that individuals tend to be risk averse when options are presented in a gain frame and risk seeking when options are presented in a loss frame, is an example of how contextual influences affect decision making (Tversky and Kahneman, 1981).

Two recent studies highlight the flexible role of testosterone in decision making (Boksem et al., 2013; Dreher et al., 2016). In a modified Ultimatum Game, testosterone increased both punishment and reward of proposers, depending on proposers’ offers. Using the Trust Game, Boksem et al. (2013) showed that testosterone modulated behavior depending on whether participants played the investor or the trustee role. Findings from these two studies support the hypothesis that testosterone flexibly influences decision-making in scenarios that involve multiple social actors. A gap in the literature is whether the observed context-dependent effects of testosterone on decision-making also extend to individual (vs. social) scenarios where contextual influences are related to how decision options are presented. This research question is particular relevant when we consider that many daily decisions happen in these individual scenarios.

**FIGURE 1:**
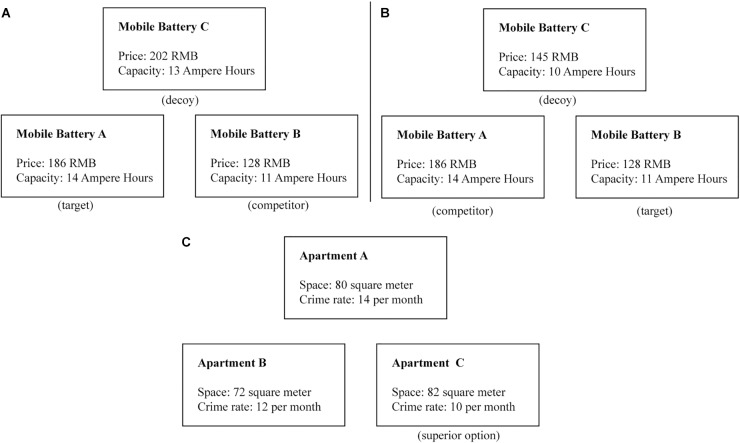
**(A)** The product on the lower left is the target, the product on the lower right is the competitor, and the product on the top is the decoy. **(B)** The product on the lower right is the target, the product on the lower left is the competitor, and the product on the top is the decoy. **(C)** A catch trial example, the product on the lower right is clearly superior to the two other products.

One way to address this gap in the literature is to test whether and how testosterone modulates decision-making when an asymmetrically dominated decoy option is introduced in a choice set. Decoy effect refers to the phenomenon that adding a new option to an existing set of options highlights the superiority of one of the existing options, shifting individuals’ preference toward that option (Huber et al., 1982). In other words, a decoy option is an additional option that is worse than one of the existing options, the dominating option, which, as a result, becomes the most attractive one. For example, as depicted in Figure 1A, a decision maker might be undecided about mobile battery A or mobile battery B. Mobile battery A has a larger capacity but is a bit more expensive, while mobile battery B has a lower capacity but is a bit cheaper. This impasse can be resolved by introducing option C, which acts as a “decoy.” Option C is better than option B in terms of capacity, worse than option B in terms of price, and, more importantly, worse than option A on both attributes (i.e., price and capacity). Thus, option C is the asymmetrically dominated decoy option, which boosts decision makers’ preference toward option A, the dominating option. Option B can become the dominating option if a different decoy option is introduced, as shown in Figure 1B. The decoy effect has been found in various domains of decision-making, including motor planning (Farmer et al., 2015), marketing strategy (Ariely, 2009), and behavioral nudge (Li et al., 2018). Here, we tested how testosterone modulated decision making in a decoy paradigm.

## Materials and Methods

### Participants

Sixty-three healthy males (mean age = 21.3 years, *SD* = 1.5, age range = 19–26) at Shenzhen University, China, were recruited through advertisement. Participants were screened over the phone and excluded if they were taking any psychotropic medication and had a history of psychiatric or neurological disorders. We tested only men because the dosing and pharmacokinetics of a single dose of Androgel used in the study have been established for men only (Eisenegger et al., 2013). Participants were instructed to abstain from alcohol, caffeine intake, and smoking for 24 h before the testing session. The study was conducted in accordance with the Declaration of Helsinki and was approved by the Shenzhen University Medical Research Ethics Committee. Written informed consent was obtained from all participants.

### Testosterone Administration

The study used a double-blind, placebo-controlled, between-subjects design. All sessions started at 13:00 and took about 4.5 h to complete. Participants in the testosterone condition received a single dose of testosterone gel, containing 150 mg of testosterone [Androgel]. Participants in the placebo condition received a colorless hydroalcoholic gel. In both conditions, a male research assistant, who was blind to the purpose of the study, applied the gel to participants’ shoulders and upper arms. The experimenter was unblinded after the data analysis was carried out. Due to the established time lag of 3 h for behavioral effects following testosterone gel application in healthy males (Eisenegger et al., 2013; Carré et al., 2015), the choice task commenced 3 h post-dosing. Participants also completed two additional decision-making tasks, which are not reported here.

### The Decision-Making Task

Participants completed a decoy decision-making task, which was adapted from Farmer et al. (2017). The task was programmed using E-Prime (version 2.0; Psychology Software Tools, Inc., PA, United States). Participants were presented with 10 pairs of products. Products in each pair were different on two attributes (e.g., Figures 1A,B). Each pair was presented twice, once with a decoy that targeted one product (Figure 1A) and once with a decoy that targeted the other product (Figure 1B); in addition, on six catch trials, one of the three products was clearly superior to the two other products (see Figure 1C). All three products (i.e., target, competitor, and decoy) were presented on each trial along with text describing their attributes. Screen locations were randomized in each trial. Using the computer keyboard, participants had to indicate their preferred product. No time constraint was imposed on choice selection. Trial order was randomized, with the only constraint that all pairs of products (and decoy) had to be presented once before being presented a second time (with a different decoy).

### Data Analysis

Participants’ choices were assigned to one of four categories (see also, Farmer et al., 2017). Consistent choices were those in which participants chose the same option when presented with the same pair of products, regardless of the decoy. Target choices were those in which participants chose the option targeted by the decoy (i.e., dominating option) (e.g., mobile battery A in Figure 1A). Non-target choices were those in which participants chose the option that was not targeted by the decoy in both presentations (e.g., mobile battery B in Figure 1A). Decoy choices were cases in which the participant chose the decoy on one or both presentations of a given product pair. Proportions in each category were calculated and compared as a function of the experimental condition (i.e., testosterone vs. placebo) using independent-samples *t*-tests.

## Results

**FIGURE 2:**
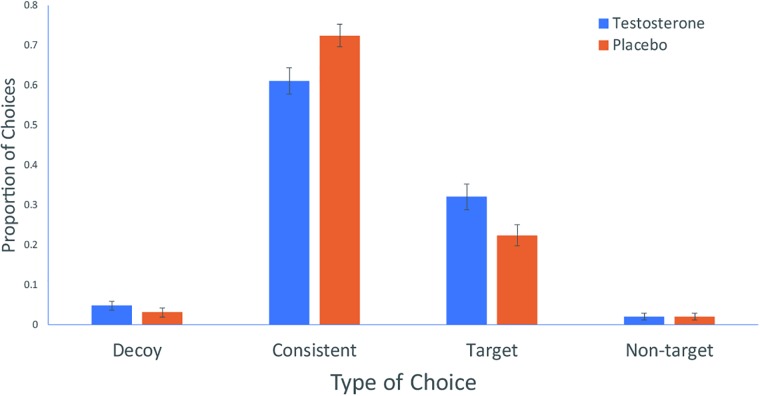
Mean proportion of choices among four possible types of choices. Error bars represent standard errors of the mean.

We excluded five participants who exhibited excessive error rate (>50%) in catch trials (i.e., failing to choose the product that was clearly superior to the other two products). As shown in Figure 2, participants in the testosterone condition (*M* = 61.03%, *SD* = 17.80%) made less consistent choices than participants in the placebo condition (*M* = 72.41%, *SD* = 15.27%), *t*(56) = −2.613, *p* = 0.012, *d* = 0.688, 95% confidence interval (CI) = [−0.201, −0.027]. Testosterone administration also increased the proportion of target choices [*t*(56) = 2.308, *p* = 0.025, *d* = 0.602, 95% CI = (0.013, 0.180); testosterone (*M* = 32.07%, *SD* = 17.40%) vs. placebo (*M* = 22.41%, *SD* = 14.31%)]. Participants in the testosterone and placebo conditions did not differ in terms of non-target choices (*M* = 2.10%, *SD* = 4.91% vs. *M* = 2.10%, *SD* = 4.12%, *p* > 0.1) and decoy choices (*M* = 4.83%, *SD* = 6.34% vs. *M* = 3.10%, *SD* = 6.04%, *p* > 0.1). The same analyses were re-run including the five participants who exhibited excessive error rate, and the same pattern of results emerged, with participants in the testosterone condition making more target choices than participants in the placebo condition [*t*(61) = 2.643, *p* = 0.010, *d* = 0.665, 95% CI = (0.025, 0.182), testosterone (*M* = 31.61%, *SD* = 16.95%) vs. placebo (*M* = 21.25%, *SD* = 14.09%)].

In a set of ancillary analyses, differences in reaction time (RT) were compared as a function of the experimental condition. For these analyses, we excluded trials in which RTs exceeded by three standard deviations participants’ mean RT. Following this procedure, 1.23% of the total data points were excluded. RT values were log-transformed in order to achieve normality. Participants in the testosterone condition (*M* = 11,501 ms^1^, *SD* = 5,007) and placebo condition (*M* = 9,800 ms, *SD* = 3,859) did not differ on the RT, *t*(56) = 1.342, *p* = 0.185.

## Discussion

Using a decoy paradigm, we found that testosterone administration reduced consistency in decision-making and increased the selection of dominating options (i.e., decoy effect). These findings are in agreement with previous work on the context-dependent nature of testosterone effects on social decision-making (Boksem et al., 2013; Dreher et al., 2016). Notably, the present study, which employed a decoy paradigm that did not involve other individuals, extends the existing literature on the flexible effects of testosterone on decision-making in social scenarios to individual scenarios. The present finding suggests that testosterone promotes behavioral flexibility by fine-tuning behaviors in response to the environment, a hypothesis recently corroborated in a correlational study testing the association between endogenous levels of testosterone and behavioral flexibility using a stimulus-outcome reversal learning paradigm (Diekhof and Kraft, 2017).

Our findings can be read in light of the dual-process theory, according to which two thought processes characterize human decision-making (Kahneman, 2011). The first process consists of rapid, automatic, emotional, and intuitive processes (i.e., System 1), while the second process is characterized by slow, effortful, and deliberate processes (i.e., System 2). Recent work on the decoy effect showed that individuals with a greater proclivity to intuitive reasoning (i.e., System 1) were more likely to be influenced by a decoy. Similarly, Pocheptsova et al. (2009) found that cognitive load, which impairs deliberation and increased reliance on intuitive processing, exacerbated the decoy effect. The present finding is also consistent with previous research showing that testosterone shifts the balance between System 1 and System 2, making individuals more reliant on intuitive decision-making. Another study showed that testosterone administration amplified emotional influences (i.e., anticipatory regret) on decision making and increased affective sensitivity to decision outcome (Wu et al., 2018). Taken together, these findings suggest that testosterone promotes intuitive and automatic judgment in human decision-making.

## Author Contributions

JL, HL, and YW developed the concepts for the study. JL and YW collected the data. JL and YW analyzed the data. All authors contributed to the manuscript and approved the final version of the manuscript for submission.

## Conflict of Interest Statement

The authors declare that the research was conducted in the absence of any commercial or financial relationships that could be construed as a potential conflict of interest.

## Footnotes

1 Note that the descriptive statistics of RTs are not transformed.
